# Building Bridges: Developing and Implementing a New Transition to Adult Care Program for Youth with Complex Healthcare Needs at a Canadian Children’s Hospital

**DOI:** 10.3390/children12081043

**Published:** 2025-08-08

**Authors:** Sara Santos, Julia Orkin, Dara Abells, Brooke Allemang, Bianca Arenas Rodriguez, Kimberly Colapinto, Nora Constas, Mackenzie Heath, Megan Henze, Tomisin John, Robyn Lippett, Susan Miranda, Joanna Soscia, Jessica Teicher, Donna Thomson, Jennifer Tyrrell, Eryn Vandepoele, Karla Wentzel, Darryl Yates, Eyal Cohen, Alène Toulany

**Affiliations:** 1 SickKids Research Institute, Child Health Evaluative Sciences, Toronto, ON M5G 0A4, Canada; sara.santos@sickkids.ca (S.S.); julia.orkin@sickkids.ca (J.O.); brooke.allemang@sickkids.ca (B.A.); bianca.arenasrodriguez@sickkids.ca (B.A.R.); tomisin.john@sickkids.ca (T.J.); joanna.soscia@sickkids.ca (J.S.); eyal.cohen@sickkids.ca (E.C.); 2 Complex Care Program, Division of Paediatric Medicine, The Hospital for Sick Children, Toronto, ON M5G 1X8, Canada; kimberly.colapinto@sickkids.ca (K.C.); susan.miranda@sickkids.ca (S.M.); 3 Surrey Place, Toronto, ON M5S 2C2, Canadamegan.henze@surreyplace.ca (M.H.); 4 The Office of Patient, Family, and Community Engagement, The Hospital for Sick Children, Toronto, ON M5G 1X8, Canada; 5 Factor-Inwentash Faculty of Social Work, University of Toronto, Toronto, ON M5S 1V4, Canada; 6 Department of Adolescent Medicine, The Hospital for Sick Children, Toronto, ON M5G 1X8, Canada; nora.constas@sickkids.ca (N.C.); mackenzie.heath@sickkids.ca (M.H.); robyn.lippett@sickkids.ca (R.L.); jennifer.tyrrell@sickkids.ca (J.T.); eryn.vandepoele@sickkids.ca (E.V.); karla.wentzel@sickkids.ca (K.W.); 7 PITCare Patient and Caregiver Advisory Committee, SickKids Research Institute, Child Health Evaluative Sciences, Toronto, ON M5G 0A4, Canada; thomsd5@mcmaster.ca; 8 Brain & Mental Health Services, The Hospital for Sick Children, Toronto, ON M5G 1X8, Canada; darryl.yates@sickkids.ca

**Keywords:** transition, youth, medical complexity, multi-morbidity

## Abstract

**Background/Objectives**: Transitioning from pediatric to adult services can be challenging for youth with complex chronic health conditions, especially those with multi-morbidity. These youth often require extra coordination and support during this phase of their healthcare journey. Building upon existing provincial and national initiatives for transitions from pediatric to adult healthcare services, we have developed a hospital-wide program within one of Canada’s largest children’s hospitals that incorporates an integrated care model aimed at better serving these patients and improving outcomes. **Methods**: Guided by provincial quality standards, an environmental scan and knowledge user engagement were conducted to develop the program, followed by an implementation phase, where the model was piloted. Ongoing learnings from the pilot continue to inform program implementation and evaluation. **Results**: The Transition to Adult Care (TAC) program offers disease-agnostic care to youth with complex needs for 1–3 years, including 1-year post-transfer, addressing the fragmentation of care across multiple services, organizations and providers. Our interdisciplinary team works in partnership with youth and caregivers to deliver transition navigation, easing the burden on patients and families by tailoring transition supports to each individual youth and caregiver. Preliminary data from the pilot revealed a lack of awareness about transition resources and timelines; however, with early engagement and flexible support beyond age 18, youth were able to complete their transition successfully. **Conclusions:** The TAC program demonstrates a systems-level approach to improving transition to adult care for youth with complex health needs by integrating individualized support, cross-sectoral collaboration, and continuous quality improvement. Early engagement, flexible post-transfer support, and close partnership with youth, caregivers, and providers are key to facilitating transition. These learnings can inform broader implementation efforts and help address persistent gaps in transitional care across healthcare systems.

## 1. Introduction

Transition to adult care is defined as a purposeful, planned movement from child to adult-oriented healthcare and social services [1], with a successful transition being characterized by early preparation, individualized transition planning, and support pre- and post-transfer to adult healthcare services. Transfer represents a one-time event during the process of transition, when the responsibility for care shifts officially from a pediatric to an adult care provider or team [2]. In Canada and many other countries, transfer typically occurs at age 18 but can take place between the ages of 16 and 19, depending on the region and type of health service involved. While some youth and caregivers navigate this transition smoothly, those with complex chronic health conditions, defined as conditions involving one or more organ systems and expected to last at least 12 months, especially those managing multiple chronic conditions (i.e., multi-morbidity), often require specialized care and extra coordination and support [3,4]. Youth lacking adequate transition support are at higher risk for discontinuity in care, poor treatment adherence, and increased acute healthcare utilization in young adulthood [5,6,7,8,9]. This risk is particularly relevant for the 15–18% of youth in North America living with a chronic health condition. Accordingly, ensuring effective transition to adult care has become a critical component and recognized priority within adolescent healthcare [3,5].

Over the past several decades, Canada has made meaningful strides in developing national and provincial guidelines aimed at improving transition policies and practices across healthcare organizations. In 2022, the Canadian Paediatric Society outlined key elements of a successful transition for youth with special healthcare needs and provided recommendations to integrate into clinical, research and advocacy work [1]. The Canadian Association of Pediatric Health Centres also convened a multi-knowledge user community of practice to develop a comprehensive national framework, complete with a repository of tools and resources to help support organizations and those responsible for delivering transitional care [6]. Within Ontario, Canada’s most populous province, quality standards were developed for transition in collaboration with healthcare professionals in both pediatric and adult care, youth, and caregivers across the region. These standards outline key domains for providing high-quality transitional care [7]. Yet, despite the publication of these guidelines, significant barriers remain to implementing and sustaining effective transition interventions for youth with chronic health conditions, especially those with multi-morbidity [8].

A recent environmental scan of the Canadian landscape reveals that transition programs are limited by a lack of provider engagement, awareness, and understanding of the holistic, multifaceted approaches and supports needed for a successful transition [8]. Much of the current transition research focuses on disease-specific interventions that mainly address specialist-to-specialist transitions while overlooking broader health, psychosocial, and environmental factors. This is especially relevant considering the prevalence and elevated risk of comorbid mental health conditions for youth living with chronic health conditions [9,10,11,12]. Health systems often struggle with resource constraints and limited funding for sustainable transition programs. Many interventions are only transiently available, typically funded by research or philanthropic sources, and most focus on the pediatric components of transition such as transfer preparation, education, and/or supporting transition readiness [8,13]. Much of the advocacy and research in transition is primarily driven by the pediatric community, despite system-level recognition of the importance of primary care providers (PCPs) and need for continuity beyond the age of transfer (e.g., age 18) [14]. The role of PCPs in transition is not well defined, despite evidence showing the association with improved uptake of adult services and continuity of care in young adulthood [15]. This results in interventions being developed and implemented with insufficient transition support matched on the adult side, leading to young adults “falling through the cracks”. The timing of transition also coincides with potentially stressful transitions in other areas of young people’s lives, such as moving out of the family home, starting post-secondary education, and entering employment. To bridge these gaps, effective transition interventions must be locally adapted to reflect national recommendations, account for regional variation in service delivery, holistically address youths’ health and psychosocial needs, and be supported by sustainable infrastructure that includes clearly defined roles for PCPs and adult healthcare providers [8].

The development of new quality standards for transition care set a foundation for addressing disparities across chronic health conditions and guiding quality improvement efforts in transition [7]. Using these standards as the pillars of our model of care, our objective was to design, evaluate, and scale an intensive Transition to Adult Care (TAC) program at The Hospital for Sick Children (SickKids) in Toronto, Ontario, to support youth with physical, mental and/or developmental complex chronic health conditions at higher risk for poor transition outcomes due to medical and psychosocial complexity. This paper outlines how Canada’s largest children’s hospital established a comprehensive, organization-wide transition program and strategy. This approach leveraged existing partnerships and funding opportunities and developed collaborations with partners within the organization, external knowledge users, as well as patients and families.

## 2. Materials and Methods

### 2.1. Program Development

#### 2.1.1. Environmental Scan

The Hospital for Sick Children (SickKids) is a free-standing quaternary care centre offering highly specialized and experimental care for rare and complex diseases not available at tertiary care centres across Canada and internationally. This pediatric hospital with 350 inpatient beds and over 290,000 ambulatory visits per year, serves approximately six million children and adolescents in Southern Ontario. In recent years, philanthropic support for rare diseases and an organizational focus on precision child health have enabled teams to advance care for populations with highly specialized and unique care needs.

A recent environmental scan of programs locally, nationally and in the literature conducted by our team revealed a notable gap in transition programs that address the unique needs of youth with complex, chronic health conditions, multimorbidity, and/or rare diseases. Our aim for establishing the TAC program was to provide transition support to youth with the most complex needs, who lacked a clear transition pathway to adult care or who faced barriers related to structural and social determinants of health. Socioeconomic barriers, along with a lack of social support, have been identified as risk factors for non-adherence to continued medical care following transfer to adult services [16]. Additionally, a scoping review identified gender, race, and ethnicity as social and structural determinants associated with inequities in transition outcomes, and the results highlighted a critical lack of studies on structural barriers in transition [17]. Due to the high risk of poor health outcomes following transition, supporting equity-deserving youth and their caregivers in navigating the transition journey is essential.

A fragmented, disease-specific approach to transition care at SickKids has contributed to poor integration into routine clinical workflows, limited standardization, and inconsistent application of best practices. While some local programs excel in transition, there is pervasive inequity in access to high quality transition care, often leaving the most vulnerable populations underserved. Youth with medical complexity, a subgroup of the population with special healthcare needs, face unique challenges. Significant caregiver involvement is often required through transition and into adulthood due to a lack of familiarity among adult providers with childhood-onset and/or rare pediatric conditions, a high proportion of co-existing intellectual and/or neuromotor disabilities, and the dependence on medical technology [18]. In response, the TAC program was intentionally designed to provide comprehensive, individualized, and holistic transition care to youth and families with the highest healthcare needs. This includes two primary groups: (1) youth working toward autonomy in their health care decisions and management; in their case, transition support is provided directly to the adolescent; and (2) youth with moderate to profound intellectual and/or neuromotor disabilities, who rely on caregivers for decision-making and medical management; in their case, transition support is directed primarily to the caregivers. This distinction reflects the reality that both groups face heightened risk for poor outcomes, yet have different needs during transition that are often overlooked. For the TAC program, a governance structure was established that incorporates hospital leadership, individuals with lived experience, as well as community and healthcare leaders, to ensure the model remains responsive to the needs of patients and their families over time.

#### 2.1.2. Knowledge User Engagement

The transition to adult services requires collaboration and integration with the adult sector, primary care, and patients and families. For this reason, our program places considerable emphasis on ensuring the engagement of all relevant knowledge users. At the program’s inception, a Patient and Caregiver Advisory Committee and an Adult Subspecialist Advisory Panel were established. Our members have contributed to the design and evaluation of our model and offer ongoing support in the iterative development of the program through regular consultation with leaders and clinicians involved in program delivery. This helps to ensure that our intervention is tailored to the needs of youth and their families, impactful, meaningful, and sustainable.

Primary care is the cornerstone of the medical home model. Engagement with PCPs is critical to ensure continuity of care, reduction in fragmentation of care, and optimizing health outcomes through an integrative systems-level approach. We established relationships with team-based PCPs early in our design phase as part of building the most effective transition solution. We also worked with the primary care leaders that report to our provincial health ministry to identify regional approaches in building community and primary care capacity through education, knowledge transfer and capacity building to better prepare community and PCPs as they support transition-age patients and their families during the transition period and beyond.

Local pediatric healthcare providers were also engaged through a series of presentations and knowledge-sharing sessions throughout the hospital to raise awareness about the TAC program and better understand their transition needs. Engagement with these groups was complemented by other knowledge mobilization activities such as pop-up information booths in common areas, conference presentations locally and internationally, websites featuring patient stories, and program pamphlets and posters.

#### 2.1.3. Evaluation Plan

We performed an environmental scan to identify a robust set of measures that are indicative of a successful transition and consulted with patients and families to determine which were most important to evaluate [19]. The selected measures were also informed by Ontario Health’s Quality Standards for transition [7], which highlight the following key areas of foci: (1) early identification and transition readiness, (2) information-sharing and support, (3) transition planning, (4) coordinated transition, (5) introduction to adult services, and (6) transfer completion. All of our outcomes address each quality standard as outlined in Table 1.

We set out to evaluate the TAC program through a phased approach, beginning with a small-scale pilot, followed by research studies with experimental designs [31]. Following institutional research ethics board approval, informed consent was obtained from study participants. Study data are kept confidential by deidentifying information, and all files will be maintained on a password-protected secure server according to institutional policies.

The pilot helped us assess the effectiveness of the care model itself (i.e., activities/workflow, caseload, and staff functions of the clinical team) and the outcome measures chosen. Clinicians involved in program delivery, researchers, and program leaders regularly engaged in Plan–Do–Study–Act (PDSA) cycles to monitor implementation, identify opportunities for improvement, and guide iterative changes [32]. Youth and caregivers complete questionnaires administered by research assistants or sent automatically via REDCap, an online survey distribution and management tool, and continue to provide feedback in qualitative interview sessions on their experiences in the program. Clinicians assessed and completed tools used to capture implementation outcomes related to the provincial quality standards. The pilot phase was critical for identifying challenges, refining processes, and ensuring both the model and evaluation tools were well-aligned with the program’s goals before broader implementation.

## 3. Results

### 3.1. Pilot Phase

Between August 2023 and November 2024, a total of 15 patients, and their primary caregiver, where applicable, began receiving transition support from the TAC program. The pilot generated several key insights that informed program refinement and expansion. The pilot underscored the importance of youth and caregiver engagement in transition and their sustained adherence to the TAC program. It confirmed the necessity for a patient-centred approach that provides disease-agnostic, individualized services to each youth and their caregiver(s), rather than applying a one-size-fits-all strategy. By demonstrating the value of the TAC team early in the program and involving youth (and caregivers, as appropriate) in decisions regarding their transition goals, we were able to secure commitment and encourage meaningful engagement. It was important that transition planning was adapted to the needs of the youth/caregiver(s). Education and navigational support provided needed to be clear and detailed, and offered through multiple modalities (in-person or virtual) and formats (verbal, written or video). Though youth and caregivers will have their own transition goals, helping them to understand and anticipate what occurs at transition, and deadlines that may affect the receipt of funding and services, is an important part of effective system navigation. However, it was important to recognize that transition is a piece of the youth and caregiver’s lives, many of the tasks expected of them are overwhelming, and youth and caregivers will act on transition items in their own time. Continuously adjusting the supports offered based on their preferences was crucial to establish trust with participants and ultimately improve their transition experience.

Establishing a new team within a new program required clearly defining the roles and responsibilities of the TAC team from the outset. Team members bring expertise in adolescent development, healthcare transitions, and system navigation. Rather than providing primary care or medical management, the TAC team focuses on supporting youth and their caregivers through the transition process. At each visit, transition needs are assessed and prioritized collaboratively. The goal is to equip youth and their caregivers with the knowledge and skills to navigate the adult healthcare system with greater confidence, while also helping adult care providers understand the unique needs of youth with complex chronic health conditions.

During the pilot, it was evident that there was a gap in understanding and awareness amongst youth and their caregiver(s) of existing transition plans and resources that were available for them. It also became clear why regular follow-up with youth after the age of 18 years is important, as many transition items are still outstanding and require support for completion. Having a team that remains affiliated with pediatric services helps to ease the grief that is felt by youth and their caregiver(s) in losing well-known teams and primary contacts, as well as familiar organizations where services are centralized in one institution.

Preliminary feedback from the pilot revealed that youth and their caregiver(s) felt the clinical team communicated well and listened to their transition-related needs and concerns. The youth also shared that they were able to express themselves openly and in a way that ‘felt less like talking to doctors.’ They greatly appreciated the flexibility of in-person and virtual appointments, as well as the comprehensive approach of the NP–social worker team. With the help of expert navigation services from the clinical team, youth and their caregiver(s) demonstrated a remarkable resilience to complete most transition items by the time the youth is 19 years, despite numerous competing priorities.

### 3.2. Transition to Adult Care Program Model of Care

The TAC program model of care was informed by Ontario Health’s Quality Standards for transition, a comprehensive environmental scan, widespread internal and external knowledge user engagement, and results from our pilot evaluation. The environmental scan was conducted both internally and externally, comparing programs across the various departments within the organization as well as across the country. No programs, either regional or national, were found to address the specific transition needs of youth with multimorbidity and/or rare diseases and social complexity. Existing programs within our hospital mostly follow a decentralized model for transition and are disease-specific. The programs receive varying levels of support, further contributing to inequities in access to much-needed resources and services.

Key components of the TAC Program include supporting transition readiness, providing developmentally appropriate information and support, collaborative development of an individualized transition care plan, care coordination, facilitating connections with primary care and adult services, and offering support post transfer (See Table 2).

Based on the gaps identified in our analysis, particularly the need for an interdisciplinary approach, and in alignment with the Quality Standards for Transition established by Health Quality Ontario, a team composed of nurse practitioners, a social worker and a nurse navigator (Registered Nurses) was created. The role of the Nurse Practitioner (NP) is to support the medical transition of the youth to ensure that all relevant information is gathered, collated, and incorporated into the youth’s transition summary. The NP also serves as a conduit and point of contact for adult subspecialists and primary care providers, and enables a warm handover of the patient transitioning from pediatric to adult healthcare system. The NP supports both the primary team at the hospital (SickKids) as well as the referring team in the adult healthcare system. This optimizes information sharing while serving as knowledge translators to support the development of capacity and knowledge among adult subspecialists and primary care providers. The nurse navigator works closely with the youth, families and caregiver(s) to ensure connection to appropriate community services and providers in areas including vocational and educational support services, community resources and funding opportunities. The nurse navigator is also responsible for the development and delivery of transition navigation education using a transition curriculum as a framework to support a more fulsome transition and expectation setting. During the work with youth and their caregiver(s), the nurse navigator is also responsible for developing and supporting youth with self-management goals. Social work supports youth and their caregiver(s) through brief mental health services, coordination of complex transition needs such as housing support, food insecurities, as well as accessing support services in the community to ensure that their mental, social and structural determinants of health are addressed as they transition to the adult health system. The role of social work is also pivotal in the customization of self-management curriculum to align with youth goals for transition and participation in self-management education visits. This team is supported by an information coordinator as well as a clinical manager. Adolescent Medicine and general pediatric physicians provide clinical consultation and support.

Youth and/or caregivers participate in approximately five to seven visits, either in-person or virtually, over a 2 to 3-year period starting at age 16 to 17. Visits are mostly virtual, preferred by youth and families during our pilot phase for their convenience and accessibility. Our hospital-based electronic health record (EMR) referral system has allowed for external community referrals into the program, extending patient outreach beyond SickKids’ catchment area. To date, we have enrolled over 150 participants from across Ontario towards our goal of approximately 280. At our current capacity and timelines, our team can support an intake caseload of 2–4 patients per week.

### 3.3. Knowledge User Engagement

At the outset, it was acknowledged that involving knowledge users was essential for the successful development of the TAC program. Engagement involved youth with lived experience, caregivers, pediatric and community providers in the design, implementation and evaluation phases of the program. The TAC Adult Subspecialist Advisory Panel was also established to provide recommendations from the adult care perspective for youth transiting to adult care. This panel serves as a platform for cross-sectorial collaboration, exchange of information and enables greater understanding of the gaps when transitioning youth complex, chronic health conditions to community agencies and adult care providers and subspecialty areas both within the region and across the province. Further, it provides a forum for sharing expertise related to critical gaps and barriers in transitioning to adult subspecialty services, as well as for informing sustainable implementation strategies.

**Figure 1:**
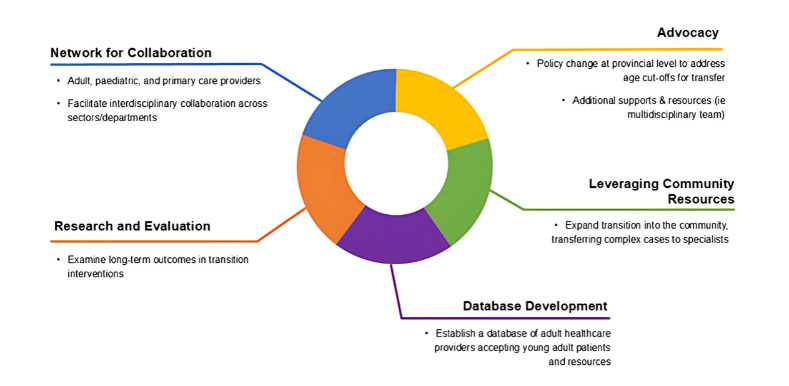
Priority areas for system-wide improvements in transition identified through knowledge user consultation.

The panel identified several key themes related to transitions in care. First, there is a clear mismatch between age-based transitions and patient readiness, with the current system often requiring premature transfers to adult services. Second, significant gaps exist in adult care infrastructure and services—many therapies routinely available in pediatric settings, such as specialty infusions, or interdisciplinary support provided by allied healthcare services, are not accessible in adult care, leading to disruptions in continuity and challenges in managing complex or rare conditions. Third, there is a lack of integration between adult, pediatric, and primary care providers, underscoring the need for improved interdisciplinary collaboration across sectors and departments. Finally, systemic and administrative barriers persist, leaving clinicians to manage strategic and operational issues without adequate structural or policy support. In response, the panel identified several areas of focus (Figure 1) for building a more effective transition network. These include: Developing a collaborative network that connects providers across the care continuum; Expanding community-based care models and strengthening referral pathways to specialists for complex cases; Establishing a database of adult care providers willing to accept transitioning patients; Advancing advocacy efforts to influence provincial policy around age cut-offs and to secure additional supports such as interdisciplinary teams; and Prioritizing research and evaluation to better understand long-term outcomes and the effectiveness of transition interventions.

The goal of the Adult Subspecialist Advisory Panel was to improve outcomes for youth while strengthening community capacity and promoting knowledge exchange. As the committee evolved, it became a valuable platform for surfacing system-level barriers, generating collaborative solutions, and fostering alignment across care settings. These discussions also brought in perspectives from external knowledge users—many of whom had limited experience with this patient population—helping to broaden understanding and highlight practical needs. Additionally, the growing network of subspecialists presents an opportunity to build stronger cross-sector relationships and ensure more coordinated support during transitions in care.

## 4. Discussion

Transition interventions have typically been driven by pediatric health systems and often fail to achieve long-term success due to insufficient consideration of systemic issues and holistic care needs. The TAC program, however, takes a broader system approach, acknowledging that successful transition requires collaboration and coordination between sectors like pediatric and adult systems, and close partnership with primary care. This presented a considerable challenge during the initial development phases of the program, as our influence as pediatric providers over adult and primary care, as well as health policy, is limited. By collaborating closely with partners across various sectors, we have begun to secure support from all levels to ensure shared responsibility and commitment to transition within our healthcare system. The purpose of our work with the TAC Adult Subspecialist Advisory Panel was to establish a systems-level approach to identifying care gaps and designing more integrated, patient-centred transition pathways. Additionally, the organizational support from our hospital, the complementarity of the precision child health hospital strategy, as well as interest from our government payer, have been instrumental in developing a model that is aligned with local healthcare priorities and youth and caregiver(s) needs.

We have intentionally developed the TAC program to be iterative in nature, applying quality improvement methods at the outset to gain feedback from youth, families and providers, and learn as we implement cyclical changes. The flexibility in this approach allows us to refine processes and procedures in real-time, making the model more attentive to the needs of youth, caregivers and providers. While we initially outlined a model of care consisting of four to six visits, in practice, patients often require additional support. Moreover, given that each youth’s transition needs and experience is unique, our team needed to be dynamic, adapting supports to meet varying constellations of complexity and often requiring individualized strategies to address diverse medical, psychosocial and systemic challenges. A challenge with this model is determining the appropriate clinician to address particular aspects of transition, given that all team members have the ability to carry out many of the same tasks. While the broad skillset of the clinical team is a strength, their interdisciplinary expertise brings inherent overlap in skills and knowledge, presenting difficulties when defining roles and responsibilities. To address this, we conducted regular discussions with the team to delineate responsibilities and ensure program efficiency. This also included defining the roles of external care teams, as well as primary care, as we work to understand each provider’s role and capacity to carry out tasks in each youth’s transition.

While the program offers transition support for youth at the highest risk for poor transition outcomes, we were required to work within resource constraints and clearly define participant eligibility criteria to ensure the delivery of a targeted intervention that addressed the needs of individuals with multiple complex chronic health conditions. We acknowledge that this decision excludes certain youth with neuro-developmental conditions, such as intellectual disability and/or cerebral palsy, who don’t have substantial medical comorbidities, a reliance on multiple specialists, fragility or dependency on medical technology. As with many program developments, we also encountered greater than anticipated staff turnover, leading to more time spent hiring and orienting new staff to both the institution and program. Additionally, we initially faced challenges in recruiting youth, despite frequent hospital visits, as their providers are often focused on patients’ acute medical needs rather than transition or are unaware of the program. To address this, we have identified eligible participants using our hospital’s EMR system, and proactively informed their providers about the program, and are exploring the development of a referral pathway where youth and/or their caregiver(s) can self-refer. The TAC program eligibility criteria reflect a need to prioritize youth with complex medical and psychosocial challenges to maximize the intervention effect. As the program evolves, we will continue to reassess eligibility criteria to ensure we are reaching the most at-risk youth and aim to disseminate our findings for widespread knowledge mobilization, allowing for replication and adaptation of the model.

## 5. Conclusions

The development and implementation of the TAC program offers valuable insights for other healthcare settings seeking to address transition gaps for youth with complex needs. A collaborative, interdisciplinary, and systems-level approach, grounded in continuous feedback and engagement with youth, caregivers, and providers, proved essential for building a responsive and sustainable model. These learnings highlight the importance of local adaptation, cross-sector partnerships, and flexible infrastructure to support meaningful and individualized transitions. Still, further research is needed to address how an intervention like the one described can be incorporated into the adult care system, as we recognize that the skills needed for successful integration and navigation in adult care are unlikely to be achieved by the time they leave the TAC program. The TAC program serves as a framework for health systems aiming to improve continuity of care and outcomes during this critical developmental period.

## Figures and Tables

**Table 1:** Mapping of TAC Program Transition Outcomes to Ontario Health Quality Standards.

Ontario Health Quality Standard	Outcome	Example of Measure/Indicator
Early Identification and Transition Readiness	Transition readiness	Transition Readiness Assessment Questionnaire
	Self-management	Patient Activation Measure ^®^ [for youth] [20], Family Empowerment Survey [for caregivers] [21]
	Patient self-efficacy	General Self-Efficacy Scale
Information Sharing and Support	Utility of care planning tools	Family Experiences With Care Coordination-16,17 [22]
	Education and resources	Youth and/or caregiver receipt of counselling and resources on needs-based services and supports
	Satisfaction with transition healthcare	Larsen Client Satisfaction Questionnaire [23]
Transition Plan	Co-creating a written transition plan with youth and caregiver(s)	Family Experiences With Care Coordination-18 [22]
Coordinated Transition	Coordination of care between and among providers and families	Family Experiences WithCare Coordination-8a,8b and 5 [22]
Introduction to Adult Services	Collaborative transitions meeting	Participation in a joint transitions meeting including pediatric providers, and key adult healthcare providers and the patient, and caregiver(s)
	Transfer initiation	Time to first adult subspecialty care visit
Transfer Completion	Transfer to continuous primary care	(a) Modified Bice–Boxerman Index [24] (b) Usual Provider of Care Index [25]
	Transfer to continuous subspecialty care	Modified Bice–Boxerman Index [24]
	Successful transfer	Attendance of the first appointment with a primary care and/or subspecialty care provider within 6 to 12 months post-transfer
Other Domains:		
Use of services	Low acuity emergency department visit	Visits to the emergency department with CTAS score of 4/5 [26]
	Emergency department use and hospitalization	Emergency Department Visits and Inpatient Days
	Technological complications	Gastrostomy, Tracheostomy and Ventriculoperitoneal shunts
	Immunization	Influenza vaccination [27]
Health-related quality of life	Caregiver fatigue	Patient-Reported Outcomes Measurement Information System Fatigue Scale [28]
	Family distress	Brief Family Distress Scale [29]
	Patient health-related quality of life and general health status ^1^	Pediatric Quality of Life Inventory (PedsQL) 4.0 Generic Core Scale Teen Report
Utility cost-effectiveness	Utility (youth/adult)	EuroQol 5-Dimension 5-Level [30]
	Utility (caregiver)	EuroQol 5-Dimension 5-Level [30]
	Incremental cost-utility ratio	Total costs of care over study interval, patient utility gains/losses and caregiver utility gains/losses
Experience with care	Overall transition experience	Qualitative interviews with youth and/or caregivers

^1^ Questionnaires will be administered based on characteristics of the patient’s health complexity and ability to complete questionnaires, as many are not suitable for complex patients with intellectual and/or neuromotor disabilities. In some cases, where available, the patient’s primary caregiver will provide a proxy.

**Table 2:** Description of Core TAC Program Activities.

Core TAC Program Activity	Description
Supporting transition readiness	Achieving transition readiness involves equipping youth and their caregiver(s) with the necessary skills to better navigate the adult healthcare system, emphasizing self-management for youth (when appropriate) to manage their health autonomously [33,34]. Central to readiness is self-efficacy, the belief in one’s ability to complete relevant activities and manage their chronic health condition(s) effectively [35]. Core principles of transition underscore the importance of routine readiness assessments and support for youths to increase their independence in managing their healthcare [2]. A comprehensive intake, focused on transition readiness and needs/risk assessment, is conducted upon acceptance into the program. Regular collaborative reviews of transition readiness are also conducted throughout to address the ongoing preparation needs of youths and their caregiver(s). The TAC program incorporates readiness preparation, self-management, and efficacy skills development into the care plan. This approach facilitates successful transitions to adult care and aims to sustain or improve health outcomes for youths with chronic health conditions.
Information sharing and support	Young people (and their caregiver(s), where appropriate) are offered developmentally appropriate information and support by the TAC team to meet their needs throughout the transition process. Information-sharing is collaborative, and healthcare providers actively seek the experience and expertise of the youth (and their caregivers, where appropriate) and incorporate it into the transition planning and shared goal-setting.
Individualized transition plan	The goal of the TAC program is to support an individualized, holistic, and coordinated transition for each patient and their caregiver (s) across multiple care settings, focused on the youth’s (and/or caregivers’) highest priority transition needs. This process includes co-developing an individualized transition plan, in collaboration with the youth and caregiver(s), to address their medical, psychosocial, and developmental needs. It also involves identifying goals and setting timelines for transition milestones. The transition plan is documented and shared within their circle of care. The clinical team oversees the refinement and maintenance of the transition plan, incorporating input from all care providers to facilitate care coordination, streamline information sharing, and consolidate multiple healthcare visits.
Care coordination	Care coordination is defined as the ‘deliberate organization of patient care activities between ≥2 participants to facilitate the appropriate delivery of healthcare services.’ [36] Successful transition encompasses the provision of continuous, well-coordinated care tailored to the developmental needs of a youth. The TAC team act as the youth’s designated most responsible provider for the transition process and engage in tasks including, but not limited to, care navigation, arranging and/or attending appointments, advocating for youth and families, and providing support throughout the transition process. This also includes facilitating information exchange with both medical and non-medical professionals central to the youth’s care (e.g., teachers, community home care providers). This process aims to foster collaborative care management among the youth’s care team while enhancing the confidence and capacity of both the youth and caregiver(s).
Connection to primary and adult care services	The TAC clinical team aims to facilitate a joint meeting with key adult providers and/or primary care before the transfer to facilitate and maintain continuity of care. This “warm handover” encourages communication among providers and active participation on the part of the youth in care decisions. Taking a proactive approach enhances the transition experience and empowers youth by recognizing their expertise in their own lives, fostering increased autonomy, independence, and confidence [37].
Post-transfer support	The TAC program extends support to youth and their primary caregiver for at least 1 year beyond the age of transfer at 18 years, thereby bridging the gap between sectors and monitoring secure attachment to adult and primary care services. Activities post-transfer include, but are not limited to, creating and following up on referrals, attending visits, facilitating connections with adult services in the hospitals and community, maintaining and updating transition documents and plans, ongoing transition progress tracking and education, addressing feelings, setting expectations, and building self-management skills (where able). Whenever possible, youth remain connected to the TAC team and are supported as they engage with and adapt to adult services, until healthcare service transitions are complete and confirmed by the youth (and their caregiver(s), where appropriate).

## Data Availability

The original contributions presented in this study are included in the article. Data is unavailable due to privacy and ethical restrictions. Further inquiries can be directed to the corresponding author.

## Abbreviations

The following abbreviations are used in this manuscript:

CMC	Children and youth with medical complexity
TAC	Transition to adult care
PCP	Primary care provider
EMR	Electronic Medical Record
